# Draft Genome Sequences of 18 *Bacteroidetes* Strains Isolated from a Human Stool Sample

**DOI:** 10.1128/mra.01218-22

**Published:** 2023-04-04

**Authors:** Basit Yousuf, Walid Mottawea, Nour Elhouda Bouhlel, Riadh Hammami

**Affiliations:** a NuGut Research Platform, School of Nutrition Sciences, Faculty of Health Sciences, University of Ottawa, Ottawa, Canada; b Department of Microbiology and Immunology, Faculty of Pharmacy, Mansoura University, Mansoura, Egypt; c Department of Biochemistry, Microbiology, and Immunology, Faculty of Medicine, University of Ottawa, Ottawa, Canada; University of Maryland School of Medicine

## Abstract

We announce the draft genome sequences of 12 *Bacteroides*, 4 *Phocaeicola*, and 2 *Parabacteroides* strains, among which was a newly isolated species, *Bacteroidaceae* bacterium UO.H1004. These isolates produce health-benefiting short-chain fatty acids (SCFAs) and the neurotransmitter γ-aminobutyric acid (GABA) in various concentrations.

## ANNOUNCEMENT

The bacterial species of the phylum *Bacteroidetes* are the predominant Gram-negative organisms colonizing the human gastrointestinal tract (GIT). *Bacteroidetes* organisms produce high concentrations of the inhibitory neurotransmitter γ-aminobutyric acid (GABA) in a strain-specific manner (1). They also produce health-benefiting short-chain fatty acids (SCFAs) in the form of acetate and propionate. GABA is presumed to provide possible beneficial effects in the brain via the gut-brain axis; the detailed mechanism is still unexplored (2–4). Also, the three metabolites play an important role in maintaining intestinal homeostasis and acting as inhibitors of proinflammatory cytokines (5, 6). Increasing evidence suggests that *Bacteroides* genera play a key role in alleviating some symptoms of different mental disorders (7). Whole-genome sequencing of these bacterial isolates will reveal the genetic mechanism underlying health benefits and their suitability as psychobiotics.

The *Bacteroidetes* species were isolated from the feces of a 29-year-old healthy female donor from Ottawa, Canada; the procedure was approved by the University of Ottawa research ethics board (certificate H-02-18-347 [29 July 2019]). The fecal slurry was serially diluted to 1E+07 in phosphate-buffered saline (PBS) and was spread on fastidious anaerobic agar with 0.5% yeast extract (FAAy) and brain heart infusion (BHI) with yeast extract, cysteine, and hemin (BHIych). Isolates were allowed to grow under anaerobic conditions (85% N_2_-10% CO_2_-5% H_2_) for 5 days. For DNA isolation, isolates were streaked on BHI agar plates, and single colonies were cultivated in BHI broth for 72 h at 37°C in an anaerobic chamber (90% N_2_-5% CO_2_-5% H_2_). Cells were collected by centrifugation, and DNA was extracted using the NucleoSpin microbial DNA kit (Macherey-Nagel, Duren, Germany) with its standard protocol. The 16S rRNA gene was PCR amplified using universal primers 8F and 1391R, and the PCR product was purified using a QIAquick PCR purification kit (Qiagen), followed by Sanger sequencing. Taxonomic attribution of the high-quality 16S rRNA gene sequences was performed with the RDP Classifier, using a nucleotide similarity cutoff value of 99% for species-level identification. Sequencing results revealed that these isolates belong to the *Bacteroides*, *Phocaeicola* (formerly *Bacteroides*), and *Parabacteroides* genera. Pure cultures of these isolates were stored in sterilized 25% glycerol at −80°C.

Illumina paired-end (2 × 151-bp) whole-genome sequencing data were generated with the MiSeq platform. Raw reads were demultiplexed, and sequencing adapters were trimmed using MiSeq Local Run Manager v3 (Illumina). The reads were then quality and length filtered using FASTQ Toolkit v2.2.5. DNA libraries were prepared with a Nextera DNA Flex kit (Illumina) according to the recommended protocol. *De novo* assembly of the Illumina reads was performed with Velvet Assembler v1.0.0 incorporated in the BaseSpace Sequence Hub (Illumina). The Rapid Annotations using Subsystem Technology (RAST) Server and automated NCBI PGAP annotation were used to annotate the assembled contigs (8, 9). Default parameters were used for all software used unless otherwise specified. Genome assembly statistics and information about the strains are summarized in Table 1.

**TABLE 1:** NCBI accession numbers, genome features, and provenance of the 18 *Bacteroidetes* isolates

Bacterial strain^*a*^	SRA accession no.	GenBank accession no.	Total genome length (bp)	Total no. of raw reads	No. of contigs	GC content (%)	*N*_50_ (bp)	Sequencing depth (×)	No. of identified protein features	No. of identified rRNA features
Phocaeicola massiliensis UO.H1001	SAMN31611827	JAQPZH000000000	4,284,320	737,240	100	42.47	100,940	51.62359	2,226	15
*Bacteroidaceae* bacterium UO.H1004	SAMN31611828	JAQPZG000000000	4,300,728	707,490	129	42.46	109,341	49.35141	2,262	16
Phocaeicola vulgatus UO.H1015	SAMN31611829	JAQPZF000000000	5,001,137	927,104	136	42.16	115,534	55.61359	2,762	6
Phocaeicola vulgatus UO.H1016	SAMN31611830	JAQPZE000000000	5,038,771	702,432	176	42.3	79,390	41.82163	2,823	6
Bacteroides cellulosilyticus UO.H1027	SAMN31611831	JAQPZD000000000	6,704,139	824,958	116	42.7	125,785	36.91561	3,357	6
Bacteroides cellulosilyticus UO.H1030	SAMN31611832	JAQPZC000000000	6,697,750	687,610	165	42.7	86,501	30.79885	3,438	5
Phocaeicola dorei UO.H1033	SAMN31611833	JAQPZB000000000	5,277,383	672,206	156	41.64	72,354	38.21246	2,876	4
Bacteroides stercoris UO.H1035	SAMN31611834	JAQPZA000000000	3,808,018	1,033,264	78	46.31	122,385	81.40172	2,106	11
Bacteroides stercoris UO.H1039	SAMN31611835	JAQPYZ000000000	3,806,727	733,410	82	46.32	91,173	57.79847	2,161	11
Bacteroides uniformis UO.H1043	SAMN31611836	JAQPYY000000000	4,503,210	694,770	51	46.41	311,955	46.28498	678	8
Parabacteroides johnsonii UO.H1047	SAMN31611837	JAQPYX000000000	4,738,509	745,708	161	45.39	66,708	47.21156	2,645	9
Parabacteroides johnsonii UO.H1049	SAMN31611838	JAQPYW000000000	4,743,270	668,724	188	45.39	52,429	42.29513	2,651	6
Bacteroides faecis UO.H1051	SAMN31611839	JAQPYV000000000	5,925,168	686,322	165	42.5	72,141	34.7495	3,087	13
Bacteroides finegoldii UO.H1052	SAMN31611840	JAQPYU000000000	4,732,446	614,060	108	42.38	106,299	38.92659	2,549	6
Bacteroides ovatus UO.H1053	SAMN31611841	JAQPYT000000000	6,660,134	778,192	232	42.02	55,912	35.05299	1,562	28
Bacteroides zhangwenhongii UO.H1054	SAMN31611842	JAQPYS000000000	5,294,039	789,796	96	41.83	140,544	44.75577	2,611	14
Bacteroides stercoris UO.H2001	SAMN31611843	JAQPYR000000000	3,983,638	762,424	94	45.89	90,608	57.41666	2,272	22
Bacteroides caccae UO.H2003	SAMN31611844	JAQPYQ000000000	5,047,099	650,004	102	41.95	132,909	38.63629	2,671	6

a All strains were collected in Ottawa, Canada, in 2022.

### Data availability.

The raw data from BioProject accession number PRJNA898401 were submitted to the NCBI Sequence Read Archive (SRA), whereas the assembled data were submitted to GenBank under BioProject accession number PRJNA922530. Experiment accession numbers are listed in Table 1.
